# Statistical Evaluation of CEB-FIP 2010 Model for Concrete Creep and Shrinkage

**DOI:** 10.3390/ma16041576

**Published:** 2023-02-13

**Authors:** Zuanfeng Pan, Haipeng Zhang, Bin Zeng, Yuwei Wang

**Affiliations:** 1 College of Civil Engineering, Tongji University, Shanghai 200092, China; 2 Central Research Institute of Building and Construction of MCC Group, Beijing 100088, China

**Keywords:** creep, shrinkage, concrete, CEB-FIP 2010, experimental database, statistical evaluation

## Abstract

An extensive experimental database consisting of 2838 shrinkage data points and 3598 creep data points is used to evaluate the accuracy of the newly proposed CEB-FIP 2010 model in predicting the creep and shrinkage of concrete structures. To study the applicability of the model for high-strength concrete in general environments, the database was developed by only retaining the test data of concrete components with the average compressive strength greater than 30 MPa and the relative humidity in the test environment less than 95%. On this basis, combined with the B3 and CEB variation coefficient methods, the paper mainly adopts the residual method to assess the accuracy of the CEB-FIP 2010 model and compare it with the previous model, CEB-FIP 1990. The influences of several properties, such as the compressive strength, the age of concrete, the relative humidity, and the component size on the prediction accuracy of these two models are further studied. The results show that for the CEB-FIP 2010 model within the time interval of 0–9000 days, 52% and 48% of the shrinkage strain residuals of the total specimens are located in the negative and positive regions, respectively, while the positive and negative regions of the CEB-FIP 1990 model account for 73% and 27%, demonstrating the CEB-FIP 2010 model has better performance in predicting shrinkage strain than the CEB-FIP 1990 model, whereas the two models have comparable accuracy in predicting creep compliance. The CEB-FIP 2010 model is more reliable for considering the effects of compressive strength, relative humidity, and age at loading on shrinkage and creep than for considering the effect of member size.

## 1. Introduction

For concrete materials, shrinkage refers to the concrete volume reduction in the hardening process of cement, and creep refers to the phenomenon that the strain of concrete increases with time under constant stress. Both creep and shrinkage are the inherent time-variance characteristics of the material itself [1], which is an important content of concrete structure design and calculation, having significant impacts on the performance of concrete structural components and systems. Creep and shrinkage affect stress distribution, widespread cracking, and excessive deformation, most of which have adverse effects on the long-term properties, usability, and durability of concrete building structures [2,3,4,5,6]. In this case, more attention needs to be paid to creep and shrinkage when designing and constructing concrete structures, particularly long-span prestressed concrete structures [7]. Because the factors influencing the concrete creep and shrinkage are extremely extensive, complex, and interrelated, coupled with the emergence of many changes, such as the material composition, production, and construction technologies, in modern concrete materials, the creep and shrinkage mechanisms of concrete are far from being fully grasped. So far, there is no widely accepted and highly accurate prediction model. In recent years, many researchers have begun to systematically study the concrete creep and shrinkage phenomena. A large number of experimental results have been statistically analyzed combined with numerical methods and computer optimization, and several mathematical prediction models have been proposed and improved [8]. Some researchers have applied the existing creep and shrinkage experimental technology to study the mechanisms, parameter influence law, and other aspects of concrete materials used in a specific structure or environment and established a corresponding creep and shrinkage calculation model. Summarizing the current research results, there are several representative creep and shrinkage prediction models, such as the ACI-209 model, CEB-FIP 1990 model, GZ model, GL2000 model, BP-KX model, B3 model, and B4 model [9,10,11,12,13,14,15]. These different models show varying shrinkage strain and creep coefficient predictions due to the different model expressions and influencing factors [16]. For example, the CEB-FIP 1990 model considers the correction factor of the mixture ratio of concrete. The ACI 209 model considers the correction factors of collapsibility, sand ratio, and air content; and the B3 model considers correction factors of the water cement ratio, cement content, sand ratio, and concrete density. In addition, according to different creep calculation methods, the above models can be divided into two types. The first type describes the overall development of creep, including the ACI 209, CEB-FIP 1990, GZ, and GL 2000 models. The second type divide creep into basic creep and drying creep, including the BP-KX, B3, and B4 models [17,18].

Compared with other concrete creep and shrinkage models mentioned above, the CEB-FIP 2010 model [19] is the latest developed model. This model is an improved version of the CEB-FIP 1990 model, which is an important basis for many concrete code creep and shrinkage calculation methods, such as JTG D62-2004 [20] and Eurocode 2 [21]. By overcoming some drawbacks of CEB-FIP 1990, the new model is more likely to be consistent with physical phenomena, the principles of thermodynamics, and linear viscoelasticity. The CEB-FIP 2010 model is applied on concretes that have a mean compressive strength extending between 20 MPa and 130 MPa and are exposed to a mean relative humidity in the range of 40 to 100% at a mean temperature in the range of 5 °C to 30 °C. The age at loading should be at least 1 day [19].

Compared with other commonly used models such as ACI 209-82, B3, and GL2000, both of the two CEB-FIP models pay more attention to the applicability of high-strength concrete. At present, there is an increasing demand for high-strength concrete in civil engineering, but the recognition of high-strength concrete’s creep and shrinkage behaviors is not sufficient. For this reason, some researchers have carried out extensive evaluation and research on the accuracy of the CEB-FIP 1990 model in predicting creep and shrinkage of high-strength concrete [22,23,24]. Some researchers [25,26] evaluated the creep coefficients of 36–90 MPa concrete calculated by the CEB-FIP 2010 model and compared them with those of the other commonly used model. However, the current research results did not fully evaluate the prediction accuracy of shrinkage and creep of the CEB-FIP 2010 model and did not consider the influence of other parameters. In general, the evaluation of the CEB-FIP2010 model is still very limited, so there is an urgent need to evaluate the applicability and prediction stability of the CEB-FIP 2010 creep and shrinkage model scientifically.

Above all, the focus of this study is motivated by a lack of evaluation studies on the CEB-FIP 2010 creep and shrinkage model against extensive experimental databases. In fact, each model has its own experimental database and may have optimal accuracy under the appropriate conditions. However, the agreement with experimental data does not represent the best agreement with the creep and shrinkage of actual engineering. High-strength concrete has lower water–cement ratio, higher compressive strength, and higher compactness compared with normal strength concrete, showing totally different creep and shrinkage behaviors. So far, the predictive accuracy of the CEB-FIP 2010 model for high-strength concrete is not clear. Therefore, the accuracy evaluation of this model based on the improved high-strength concrete database has great theoretical significance and practical value. It can compensate for the deficiency of an insufficient understanding of creep and shrinkage mechanisms of high-strength concrete at present.

Therefore, in this paper, an extensive compiled database available in the literature [22] is employed and developed for evaluation studies. The formula provided by the CEB-FIP 2010 model is adopted to determine the creep compliance and the shrinkage strain of each specimen and compare them with the experimental data. Meanwhile, combined with the B3 and CEB coefficients of variation methods [27,28], the residual method was mainly used to evaluate the model. For the contrast group, the corresponding parameters in the CEB-FIP1990 model were also calculated. On this basis, the accuracy of these two CEB-FIP models in predicting the creep compliance and shrinkage strain of high-strength concrete is further discussed.

## 2. CEB-FIP 2010 Model

Based on the principles of viscoelastic mechanics, the CEB-FIP 2010 model [19] provides a new method to evaluate the creep and shrinkage of concrete structures, makes some improvements to correct the shortcomings of the original CEB-FIP 1990 model, and introduces some additional provisions to distinguish different parts of creep and shrinkage, which leads to some aspects of the long-term behavior of concrete to be better identified. More specifically, a new formula for the total shrinkage strain is proposed. The total shrinkage strain is defined as the sum of the autogenous shrinkage and the drying shrinkage, and the formulas for these two parts with time are proposed. Moreover, similar to the GL2000 model, the CEB-FIP 2010 model [19] subdivides creep into basic creep and dry creep. In this new model, only a few parameters that are the same as those in CEB-FIP 1990 and can be used by designers are considered. The calculation method and related parameters of the CEB-FIP 2010 model [19] are provided in the following context.

### 2.1. Shrinkage

The total shrinkage strain, *ε_sh_*(*t*,*t_s_*), of concrete is the sum of autogenous shrinkage *ε_auto_*(*t*) and drying shrinkage *ε_dry_*(*t*,*t_s_*), calculated by using Equations (1)–(5).

(1) $$\varepsilon_{sh}\left(t,t_{s}\right)=\varepsilon_{auto}\left(t\right)+\varepsilon_{dry}\left(t,t_{s}\right)$$

(2) $$\varepsilon_{auto}(t)=-\alpha_{as}{\left(\frac{f_{cm}/10}{6+f_{cm}/10}\right)}^{2.5}\cdot [1-\exp(-0.2\sqrt{t})]\cdot 10^{-6}$$

(3) $$\varepsilon_{dry}\left(t,t_{s}\right)=\left[(220+110\cdot \alpha_{ds1}\right)\exp\left(-\alpha_{ds2}\cdot f_{cm})\right]\cdot \beta_{RH}\cdot {\left[\frac{\left(t-t_{s}\right)}{0.035\cdot h^{2}+\left(t-t_{s}\right)}\right]}^{0.5}\cdot 10^{-6}$$

(4) $$\beta_{RH}=\{\begin{array}{cc} -1.55\cdot \left[1-{\left(\frac{RH}{100}\right)}^{3}\right] & \mathrm{for}\;40\%\leq RH<99\%\beta_{s1}\\ 0.25 & \mathrm{for}\;RH\geq 99\%\beta_{s1} \end{array}$$

(5) $$\beta_{s1}={\left(\frac{35}{f_{cm}}\right)}^{0.1}\leq 1$$

where *t* is the age of concrete in days; *t_s_* is the concrete age at the beginning of drying in days; *α_s_*, *α_s_*_1_, and *α_s_*_2_ are three coefficients depending on the type of cement, the values of which can be found in [19]; *f_cm_* is the average compressive strength of concrete at 28 days, *RH* is relative humidity, *h* means the notional size of member defined as the ratio of twice the area of cross-section to the perimeter of the member, and *β_RH_* is a coefficient taking into account the effect of the ambient relative humidity; and *β_s_*_1_ is a calculation coefficient related to the compressive strength of concrete.

### 2.2. Creep

The creep coefficient *φ*(*t*,*t*_0_) is represented by the sum of the basic creep coefficient *φ_bas_*(*t*,*t*_0_) and the drying creep coefficient *φ_dry_*(*t*,*t*_0_) and is computed using Equations (6)–(10), given as:

(6) $$\varphi (t,t_{0})=\varphi_{bas}\left(t,t_{0}\right)+\varphi_{dry}\left(t,t_{0}\right)$$

(7) $$\varphi_{bas}\left(t,t_{0}\right)=\frac{1.8}{{\left(f_{cm}\right)}^{0.7}}\cdot \ln\left[{\left(\frac{30}{t_{0}}+0.035\right)}^{2}\cdot \left(t-t_{0}\right)+1\right]$$

(8) $$\varphi_{dry}\left(t,t_{0}\right)=\frac{412}{{(f_{cm})}^{1.4}}\cdot \frac{1-\frac{RH}{100}}{\sqrt[{3}]{0.1\cdot \frac{h}{100}}}\cdot \frac{1}{0.1+t_{0}{}^{0.2}}\cdot {\left[\frac{\left(t-t_{0}\right)}{\beta_{h}+\left(t-t_{0}\right)}\right]}^{\gamma (t_{0})}$$

(9) $$\gamma (t_{0})=\frac{1}{2.3+\frac{3.5}{\sqrt{t_{0}}}}$$

(10) $$\beta_{h}=1.5\cdot h+250\cdot {\left(\frac{35}{f_{cm}}\right)}^{0.5}\leq 1500\cdot {\left(\frac{35}{f_{cm}}\right)}^{0.5}$$

where *t*_0_ is the concrete age at the beginning of loading in days. Note that an additional coefficient (1/0.1+*t*_0_^0.2^) is introduced to modify the age of the concrete *t*_0_ accounting for the effect of cement [19]. *β_h_* is a calculation coefficient taking into account the effect of the notional size of the member and the mean compressive strength of the concrete.

Experimental studies covering various environmental conditions and time spans have shown that the CEB-FIP 2010 model is suitable for concrete that has a 28-day mean compressive strength in the range of 20 MPa to 130 MPa and was cured in an environment with average relative humidity in the range of 40% to 100% and average temperature in the range of 5 °C to 30 °C. Compared with the CEB-FIP 1990 model, the maximum compressive strength applicable to the CEB-FIP 2010 model increased from 90 MPa to 130 MPa, which indicates that the new model has the ability to predict the creep and shrinkage characteristics of high-strength concrete.

## 3. Creep and Shrinkage Test Data

Compared with the database constructed by former researchers [23,29,30], the database [22] adopted in this paper was developed by considering two additional factors: concrete compressive strength and relative humidity. Firstly, 30 MPa is the minimum concrete compressive strength limit in the database used in this paper. This is because the research focus of this paper is to verify the applicability of the model to the creep and shrinkage characteristics of high-strength concrete, and low-strength data points, which have been removed from the database, would negatively affect the model evaluation. On the other hand, creep and shrinkage have a significant impact on the long-term performance of long-span concrete structures, so prestressed concrete is usually used for construction. According to AASHTO [10], the minimum concrete compressive strength for prestressed structures is 28 MPa, but in engineering practice, this value usually exceeds 30 MPa. In this case, it is reasonable to only consider the data points about concrete with compressive strengths larger than 30 MPa in this database. The second consideration is that tests on concrete specimens exposed to the environments with 99% or 100% relative humidity, which only occur underwater, are excluded from the database. This is because the long-span prestressed concrete structures are often in service above water or ground, where the relative humidity is usually lower than 95% for the atmospheric environment. Hence, restricting the maximum relative humidity in the database to 95% is necessary. In addition, the database also includes many experiments conducted in China in comparison to previous databases.

For some experiments in the database, only the 28-day cube’s compressive strength was measured. To achieve a fair comparison, the cubic compressive strength is converted to the cylinder compressive strength. It is noteworthy that compressive strength conversion in CEB-FIP 2010 is different from that in CEB-FIP 1990. For the cylinder strength larger than 60 MPa and smaller than 110 MPa, the cube compressive strength is specified to be 15 MPa larger than the cylinder compressive strength in CEB-FIP 2010 instead of 10 MPa in CEB-FIP 1990.

### 3.1. The Shrinkage Experimental Database

For the shrinkage experimental database used in this paper, 48 sets of test data were obtained in China [31,32,33,34,35,36,37,38], and the remaining 158 sets were from other countries. Table 1 shows the distribution of three main influencing properties in 206 sets of shrinkage test data, including the strength of concrete, the relative humidity of environment, and the effective thickness of components. It can be seen from Table 1 that the number of specimens with a 28-day mean compressive strength range of 30 MPa to 60 MPa accounts for 73.7% of the total number of shrinkage tests, and this range of compressive strength is common in prestressed concrete structures. The large proportion of normal-strength concrete in the database implies that this database can represent shrinkage in common normal-strength concrete. In this database, the ambient relative humidity recorded from the shrinkage test data is mostly between 40% and 80%, accounting for 91.7% of the total number of shrinkage tests, which is similar to the environment in which the actual structure is located. In addition, most shrinkage test specimens are relatively smaller in size compared to the actual concrete components due to the limit of the laboratory space, and the effective thickness of 93.7% of the total specimens is less than 100 mm.

### 3.2. The Creep Experimental Database

For the creep experimental database used in this paper, 35 sets of test data were obtained in China [31,32,33,39,40,41], and the remaining 144 sets were from other countries. Table 2 shows the distributions of the data points over four different influencing properties in 179 total sets of creep test data, including the strength of concrete, the relative humidity of the environment, the effective thickness of components, and the concrete loading age. As shown in Table 2, the number of specimens with the concrete 28-day mean compressive strength range from 30 MPa to 60 MPa accounts for 86% of the total number of creep tests. This is similar to the situation in the database of shrinkage. In the database, 96.7% of data sets were measured in environments with 50–65% relative humidity that are comparable to the actual conditions for ordinary concrete structures. The creep experiments, similar to the shrinkage experiments, were conducted in standard indoor environments where the sizes of test specimens were smaller than those of the actual concrete members, so the effective thickness of 93.9% of the total specimens was between 25 mm and 100 mm. The concrete loading age of creep experiments in the database is mainly within 28 days, accounting for 78.8% of the total number of tests. During the construction process of an actual prestressed, reinforced concrete bridge, the loading age of concrete is uncertain, ranging from several days to hundreds of days. In addition, the construction of long-span concrete bridges includes different construction stages, and the concrete loading age of each stage is also uncertain. Therefore, the concrete loading ages of creep specimens selected in the database also include data exceeding 28 days, accounting for 21.1% of the specimens.

## 4. Model Evaluation Methods

Based on the aforementioned creep and shrinkage test database, the residual method was mainly used to assess the applicability of the CEB-FIP 2010 model. At the same time, the B3 variation coefficient and CEB variation coefficient corresponding to the model were calculated. As a commonly used, simple and clear model evaluation method, the residual method involves estimations of the difference between predictions and experiments in terms of strain or creep compliance. The basic approach is to subtract the experimental value from the prediction value calculated by the model. If the difference is positive, the shrinkage strain or creep compliance is overestimated by the prediction model, while a negative infers the opposite prediction. The value of residual (*RV*) is determined as follows:

(11) RV=P−E

In the above formula, *E* means the measured value of the experiment and *p* means the prediction value of the model. Obviously, the smaller the absolute value of the residual is, the more accurately the creep and shrinkage model predicts. Moreover, in order to evaluate the distribution of residuals over time, the specimens are separated into three different groups based on the days at loading or drying. In addition, for the purpose of quantifying the accuracy of the model, the data points of either shrinkage or creep are further categorized into two groups based on the limits of the strain residual, ±100 με for shrinkage models and ±33 με/MPa for creep model.

The analysis procedure of the B3 coefficient of variation method [27] is more complex than the other two methods. In this method, the loading time of concrete is divided into several intervals according to the logarithmic scale of time, including 0–10 days, 11–100 days, 101–1000 days, and 1101–10000 days. The calculation weight coefficient of different time intervals is determined by the number of data points in the corresponding time interval. The purpose of this is to ensure that the overall variation coefficient is not affected by data deviations within a certain time interval, thus ensuring the accuracy of the overall variation coefficient. The other evaluation method, the CEB variation coefficient method, was developed by Muller and Hilsdorf [28]. More detailed descriptions of CEB as well as B3 can be found in the literature [27,28].

## 5. Analysis Results

In this section, the CEB-FIP 2010 model is evaluated by three approaches introduced previously using the database of shrinkage and creep. For comparative purpose, the CEB-FIP 1990 model is also adopted to calculate the creep compliance and shrinkage strain of each specimen. In addition, investigations on the dependencies of the major parameters, including the relative humidity, the concrete strength, the effective thickness, and the loading age, on the accuracy of prediction of the model are also carried out.

### 5.1. Evaluation of the Shrinkage Models

**Figure 1:**
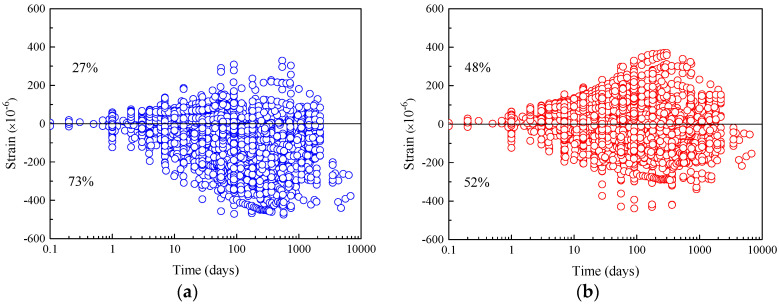
The residuals of shrinkage strain for CEB-FIP models versus time. (**a**) Residuals for CEB-FIP 1990 model. (**b**) Residuals for CEB-FIP 2010 model.

Figure 1 shows the distribution of the shrinkage strain residual of these two prediction models. As a comparison, the results calculated by the CEB-FIP 90 model in the literature [22] are also provided, as specified in Table 3. In the figure, the positive residual value indicates that the predicted value of the model overestimates the shrinkage strain. On the contrary, if the residual value is negative, it indicates that the predicted value of the model underestimates the shrinkage strain. As shown in Figure 1a, 73% of the test specimens have negative shrinkage strain residuals, indicating that the shrinkage strain of the specimens is underestimated by the 1990 model. Correspondingly, as shown in Figure 1b, the shrinkage strain residuals of 52% of the specimens are negative, while the shrinkage strain residuals of the remaining 48% of the specimens are positive, and the number of residual values on both positive and negative sides is basically the same. This indicates that the shrinkage strain calculated by the CEB-FIP 2010 model is more accurate. Furthermore, the numbers of positive and negative residuals of shrinkage strain determined by the CEB-FIP 2010 model, shown in Table 3, are approximately equal in the 0–1000-day time interval. However, the number of negative residuals (61% of total specimens) is greater than the number of positive residuals (39% of total specimens) in the time interval of 1001–9000 days, indicating that the shrinkage strains are underestimated at this phase. As for the 1990 model, most of the residual values of shrinkage strain are negative regardless of being short-term or long-term test results, which indicates that the previous shrinkage model has the risk of underestimating concrete shrinkage strain. Furthermore, the results calculated by CEB-FIP 2010 show that 65% of the total specimens exhibit shrinkage strain residuals within the range of −100 με and +100 με for 0–9000 days, better than the results of the CEB-FIP 1990 model (58% of the total specimens). The *ω*_B3_ and *V*_CEB_ of the CEB-FIP 2010 model are 59.9% and 57.0%, respectively, which are higher than those of the CEB-FIP 1990 shrinkage model (47.6% and 44.3%).

**Figure 2:**
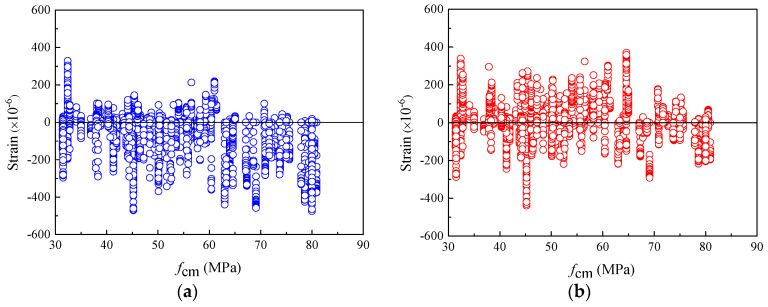
The residuals of shrinkage strain for CEB-FIP models versus *f_cm_*. (**a**) Residuals for CEB-FIP 1990 model (**b**) Residuals for CEB-FIP 2010 model.

As mentioned above, the widely accepted and applied creep and shrinkage models in practical engineering were mostly developed by strictly mathematical statistical calculation with the experimental data of normal-strength concrete. Due to the large proportion of normal-strength concrete specimens in the tests, the adaptability of most models to ordinary-strength concrete will be better. Correspondingly, the prediction accuracy for concretes with higher strength will be worse. Figure 2 illustrates the shrinkage strain residuals of the specimens with different compressive strengths *f_cm_*. Figure 2a shows that the shrinkage strains of specimens with compressive strengths greater than 40 MPa are seriously underestimated by the previous CEB-FIP 1990 model. In addition, as shown in Figure 2b, for the CEB-FIP 2010 model, the number of specimens with positive residuals is approximately equal to those with negative residuals for either normal- or high-strength concrete. For the high-strength concrete with a given *f_cm_*, such as *f_cm_* > 65 MPa, the absolute values of residuals computed using the CEB-FIP 2010 model are generally smaller than those calculated using the CEB-FIP 1990 model. This implies that the CEB-FIP 2010 model has better performance in predicting the high-strength concrete shrinkage strain.

**Figure 3:**
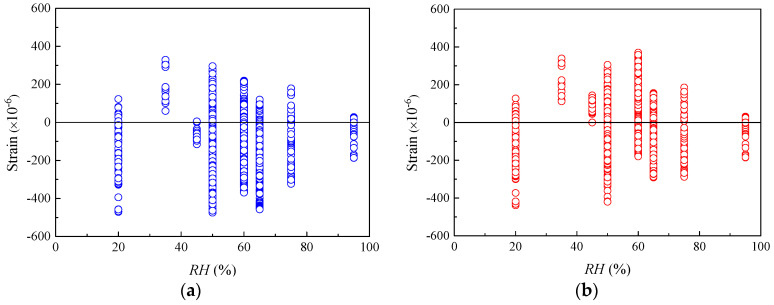
The residuals of shrinkage strain for CEB-FIP models versus RH. (**a**) Residuals for CEB-FIP 1990 model. (**b**) Residuals for CEB-FIP 2010 model.

**Figure 4:**
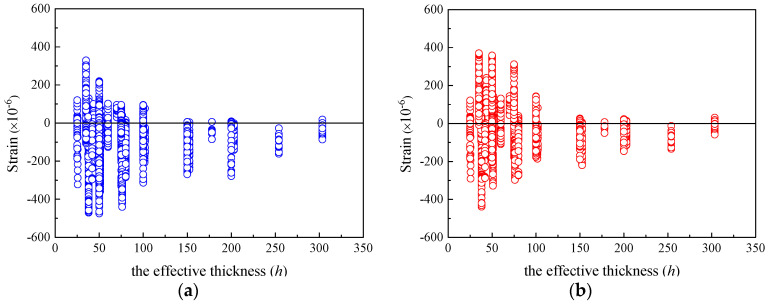
The residuals of shrinkage strain for CEB-FIP models versus h. (**a**) Residuals for CEB-FIP 1990 model. (**b**) Residuals for CEB-FIP 2010 model.

Figure 3 shows that when the relative humidity *RH* is in the range of 50–80%, the residual values of the specimens are distributed uniformly in the positive and negative regions, indicating both models can accurately predict the shrinkage strain of concrete under ordinary atmospheric humidity environments. Figure 4a depicts that the shrinkage strains of most of the specimens are underrated by the CEB-FIP 1990 model with various *h*. Figure 4b shows that the residuals computed using the CEB-FIP 2010 model distribute uniformly in the negative and positive regimes for the specimens with *h* smaller than 100 mm. CEB-FIP 2010 generally underestimates the shrinkage strains of the specimens with *h* larger than 100 mm. The absolute values of residuals for the specimens with *h* larger than 100 mm calculated using CEB-FIP 2010 are relatively small as compared to those computed by CEB-FIP 1990. Note that the effective thicknesses of actual concrete components usually exceed 100 mm, and the CEB-FIP 2010 shrinkage model is more reliable than CEB-FIP 1990 in considering the influence of components size on shrinkage strain.

### 5.2. Evaluation of the Creep Models

**Figure 5:**
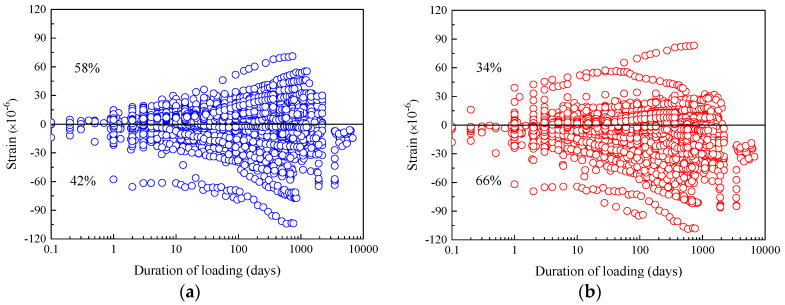
The residuals of creep compliance for CEB-FIP models versus time. (**a**) Residuals for CEB-FIP1990 model. (**b**) Residuals for CEB-FIP 2010 model.

Figure 5 shows the creep compliance residuals distribution of these two prediction models. As a comparison, the results calculated by the CEB-FIP 1990 model in the literature [22] are also provided, as specified in Table 4. For the prediction results of the CEB-FIP 2010 model, the creep compliance residuals for 66% of the total specimens are negative and the remaining 34% are positive, as can be seen from Figure 5a. In contrast, the prediction results provided by the CEB-FIP 1990 creep model are more uniform; 42% of the specimens are negative and 58% are positive. In addition, in the 0–9000-day interval, for the CEB-FIP 2010 prediction model, 88.4% of the data points with residual values are between −33 με/MPa and +33 με/MPa, which is less than the proportion of the CEB-FIP 1990 model’s predicted values in this interval (93%). Therefore, the CEB-FIP 1990 model shows better stability in predicting the creep compliance, and the CEB-FIP 2010 model underestimates the creep compliance of concrete. The calculated values of the B3 variation coefficient and CEB variation coefficient of the CEB-FIP 2010 creep model were 29.3% and 26.0%, respectively, which are equivalent to the calculated values of the CEB-FIP 1990 creep model (B3 variation coefficient is 28.0% and CEB variation coefficient is 27.5%).

It can be seen from the above analysis that although the influencing factors considered by the CEB-FIP2010 model and the CEB-FIP1990 model are basically the same, the calculation results of the two models are quite different. The reason is that the CEB-FIP2010 model has a large adjustment to the calculation formula. The model is expressed as the sum of the two coefficients of basic creep and dry creep. Furthermore, the two coefficients are expressed as the multiplication of several factors, and the corresponding concrete compressive strength and time influence factors are given.

**Figure 6:**
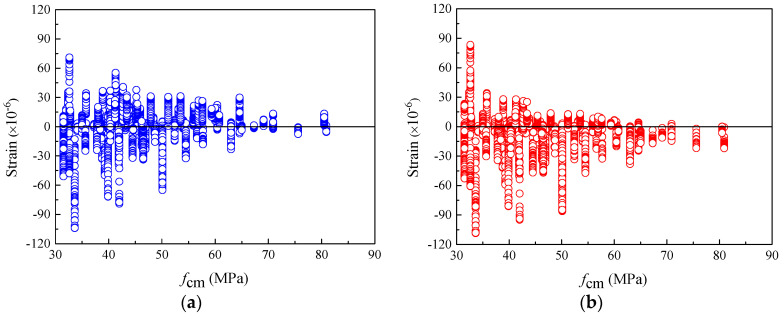
The residuals of creep compliance for CEB-FIP models versus *f*_cm._ (**a**) Residuals for CEB-FIP 1990 model. (**b**) Residuals for CEB-FIP 2010 model.

Figure 6a reveals the relationship between the creep compliance residual and the concrete compressive strength *f_cm_* for the CEB-FIP 1990 model. When *f_cm_* > 40 MPa, most of the residual data points are positive, which implies that the high-strength concrete creep compliance is overestimated by the CEB-FIP 1990 model. Considering this case, the CEB-FIP 2010 model has a large adjustment to the influence factor of concrete strength by strengthening the inverse relationship between the calculation coefficient and concrete compressive strength. For example, in Equation (10), the influence of concrete compressive strength is also considered in the calculation of the notional size influence coefficient *β*_h_. These adjustments correct the CEB-FIP 1990 model for overestimating the creep compliance of high-strength concrete. As can be seen from Figure 6b, for the CEB-FIP 2010 model, when *f_cm_* > 40 MPa, the number of data points with a positive residual value is significantly smaller than that of the CEB-FIP 1990 model. Therefore, the CEB-FIP 2010 model is more suitable for predicting the creep compliance of high-strength concrete.

**Figure 7:**
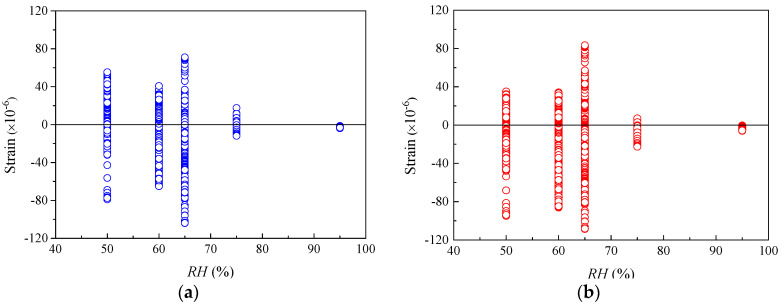
The residuals of creep compliance for CEB-FIP models versus *RH.* (**a**) Residuals for CEB-FIP 1990 model. (**b**) Residuals for CEB-FIP 2010 model.

**Figure 8:**
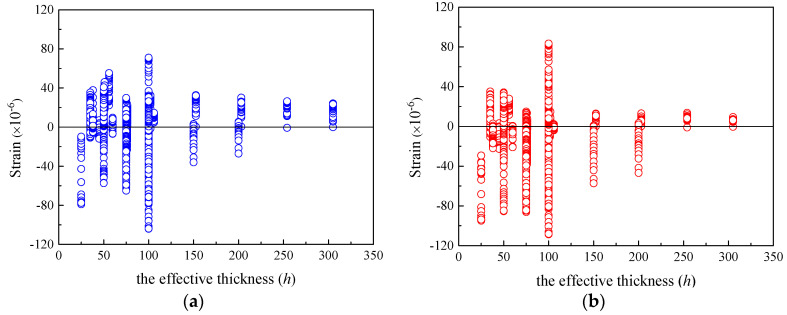
The residuals of creep compliance for CEB-FIP models versus *h.* (**a**) Residuals for CEB-FIP 1990 model. (**b**) Residuals for CEB-FIP 2010 model.

Figure 7 and Figure 8 show the distribution of creep compliance residuals versus *RH* and *h*. As is seen from Figure 7, the distributions of residuals for both CEB-FIP models lie in both regimes of positive and negative residuals. This demonstrates that the two models have comparable performance in practical applications to the concrete under environments with ordinary atmospheric humidity (50% < *RH* < 95%). Figure 8 indicates that for both models, the positive and negative residuals distribute uniformly for concrete when *h* is smaller than 100 mm. However, the residuals of the specimens with *h* larger than 100 mm are generally overestimated by CEB-FIP 1990 and underestimated by CEB-FIP 2010. Because the effective thicknesses of actual concrete members are usually larger than 100 mm, the CEB-FIP creep models may be unreliable to consider the effect of member size on creep compliance.

### 5.3. Discussion

In conclusion, the evaluation results show that the CEB-FIP 2010 shrinkage model has better performance than the CEB-FIP 1990 shrinkage model. Compared with the CEB-FIP 1990 creep model, the CEB-FIP 2010 creep model has no obvious advantages, and for the database adopted by this study, only a few shrinkage or creep tests were carried out in more than 10,000 days. However, concrete structures, especially some important infrastructure such as bridges, need to be evaluated for their expected life (usually up to 100 years). Therefore, more test data are needed to improve the current database in order to further study the predictive accuracy of the CEB-FIP 2010 model for the long-term performance of concrete.

## 6. Conclusions

The database used and developed in this study, derived from reference [22], contains a large amount of creep and shrinkage test data and has three significant features: firstly, in this database, the minimum concrete compressive strength is strictly limited to 30 MPa; secondly, the maximum relative humidity is limited to 95%; thirdly, a large number of test data from experiments carried out in China are incorporated in the database. Subsequently, the residual method is mainly used to evaluate these two CEB-FIP models based on the improved database, and the corresponding B3 coefficient of variation and CEB coefficient of variation are also calculated. The model evaluation results are valuable to the practical design of concrete structures, particularly prestressed concrete structures. The following conclusions have been drawn from this paper:

(1) For the experimental shrinkage database used in this paper, 48 sets of test data were obtained in China, and the remaining 158 sets were from other countries. For the experimental creep database, 35 sets of test data were obtained in China, and the remaining 144 sets were from other countries. The distribution of the data over several influence parameters shows that the database constructed in this paper can well-represent the real engineering environment.
(2) The calculation results provided by the CEB-FIP 2010 model show that within the time interval of 0–9000 days, 52% and 48% of the shrinkage strain residuals of the total specimens are located in the negative and positive regions, respectively, while the positive and negative regions of the residuals of the 1990 model account for 73% and 27%. The residual distribution of shrinkage strain shows that the CEB-FIP 2010 shrinkage model has stable prediction performance, while the shrinkage strain of concrete is apparently underestimated by the previous 1990 model. In addition, the shrinkage strain residuals of concrete calculated by the CEB-FIP 2010 model are evenly distributed in the negative and positive regions under various compressive strengths *f_cm_*, even for concrete with compressive strengths above 40 MPa, which means that this model provides more accurate prediction of shrinkage strain in high-strength concrete.
(3) For the residual value of creep compliance calculated by the CEB-FIP 2010 model, in the time interval of 0–9000 days, the residual values of creep compliance of 66% and 34% of the total specimens fall into the negative and positive regions, respectively, indicating that this model underestimates the creep compliance of concrete, which is similar to the evaluation results of the previous 1990 model. The CEB-FIP2010 creep model has no significant improvement in creep prediction. However, for the prediction of high-strength concrete creep compliance, the CEB-FIP 2010 model shows higher accuracy and better stability, which corrects the CEB-FIB 1990 model for overestimating the creep compliance of high-strength concrete.
(4) For concrete specimens under the conditions of *RH* varying from 50% to 80%, the predicted results by both CEB-FIP models are favorable, which implies that the two models are applicable in practical environments with ordinary atmospheric humidity. Moreover, only for the specimens with *h* smaller than 100 mm, both CEB-FIP models exhibit satisfying predictive performance. Since the effective thicknesses of actual concrete members are usually larger than 100 mm, if the influence of member size effect is considered, the prediction results of these two CEB-FIP models are not reliable.

## Figures and Tables

**Table 1:** Distribution of major parameters of the shrinkage database.

Strength (MPa)	30–40	40–50	50–60	60–70	70–81
Number of data sets	34 (16.5%)	79 (38.3%)	39 (18.9%)	32 (15.5%)	22 (10.7%)
Relative humidity (%)	20–40	40–60	60–80	80–95	—
Number of data sets	10 (4.9%)	110 (53.4%)	79 (38.3%)	7 (3.4%)	—
Effective thickness (mm)	25–45	45–70	70–100	100–305	—
Number of data sets	44 (21.4%)	79 (38.3%)	70 (34.0%)	13 (6.3%)	—

**Table 2:** Distribution of major parameters of the creep database.

Strength (MPa)	30–40	40–50	50–60	60–70	70–81
Number of data sets	50 (27.9%)	67 (37.4%)	37 (20.7%)	18 (10.1%)	7 (3.9%)
Relative humidity (%)	50	60	65	75–95	—
Number of data sets	35 (19.6%)	64 (35.8%)	74 (41.3%)	6 (3.4%)	—
Effective thickness (mm)	25–45	45–70	70–100	100–305	—
Number of data sets	22 (12.3%)	46 (25.7%)	100 (55.9%)	11 (6.1%)	—
Age at loading (days)	1–5	5–14	14–28	≥28	—
Number of data sets	17 (9.5%)	49 (27.4%)	75 (41.9%)	38 (21.2%)	—

**Table 3:** Distribution of shrinkage strain residuals for CEB-FIP 1990 and 2010 shrinkage models.

Period		0–1000 Days		1001–9000 Days		0–9000 Days	
		CEB 90	CEB 10	CEB 90	CEB 10	CEB 90	CEB 10
Residual ^a^	Number of data points	2660 (94%)		178 (6%)		2838 (100%)	
	*RV* > 0	728 (27%)	1294 (49%)	35 (20%)	70 (39%)	763 (27%)	1364 (48%)
	*RV* < 0	1932 (73%)	1366 (51%)	143 (80%)	108 (61%)	2075 (73%)	1474 (52%)
	|*RV*| < 100 με	1553 (58%)	1746 (66%)	101 (57%)	88 (49%)	1654 (58%)	1834 (65%)
	|*RV*| > 100 με	1107 (42%)	914 (34%)	77 (43%)	90 (51%)	1184 (42%)	1004 (35%)

^a^ Note that |RV| denotes the absolute value of residual.

**Table 4:** Distribution of creep compliance residuals for CEB-FIP 1990 and 2010 shrinkage models.

Period		0–1000 Days		1001–9000 Days		0–9000 Days	
		CEB 90	CEB 10	CEB 90	CEB 10	CEB 90	CEB 10
Residual	Number of data points	3353 (93%)		245 (7%)		3598 (100%)	
	*RV* > 0	1960 (58%)	1158 (35%)	135 (55%)	75 (31%)	2095 (58%)	1233 (34%)
	*RV* < 0	1393 (42%)	2195 (65%)	110 (45%)	170 (69%)	1503 (42%)	2365 (66%)
	|*RV*| < 33 με/MPa	3125 (93%)	2984 (89%)	220 (90%)	197 (80%)	3345 (93%)	3181 (88%)
	|*RV*| > 33 με/MPa	228 (7%)	369 (11%)	25 (10%)	48 (20%)	253 (7%)	417 (12%)

## Data Availability

The data presented in this study are available on request from the corresponding author.
